# Care of the Deciduous Teeth, (Extracts from a Discussion)

**Published:** 1902-03-15

**Authors:** 


### CARE OF THE DECIDUOUS TEETH.

Extracts from the discussion of a paper read at the California State Dental Association and reported in the *Pacific Gazette*.

If a child's first visit to the dentist is unaccompanied by painful treatment, it will be much easier to perform other operations when occasion requires. Parents are often to blame for the feeling of apprehension which their children exhibit on their first visit to the dentist, caused in many cases by relating and exaggerating their own experiences within the hearing of the children. —Dr. W. J. Taylor.

We are often asked by parents, "When shall I bring my children to have their teeth examined?" I tell them, "Just as soon as your child erupts its first tooth, or even sooner, should the child experience trouble in erupting the teeth. I have seen children less than three years old with the teeth hopelessly ruined. A case of this kind, a little girl not yet three years old, was brought to me the day before I left home for this meeting, who did not have a sound tooth in her head; there were five abscesses discharging pus. The mother complained that the child was unhealthy and did not grow, and she could not tell why. I informed her that she need look no further than the child's mouth for the reason, and plainly informed her that she ought to be in jail for so neglecting that child's teeth.—F. L. Platt.

A child can not develop a physical life when hindered by a mouthful of diseased teeth; much less can it develop a happy and wholesome moral life under similar conditions, and for this reason alone children's teeth ought to receive more careful attention than they are accustomed to have.—H. G. Atwater.

If, as someone has said, the education of a child should begin 400 years before its birth, it is equally reasonable to argue that the care of a child's teeth should be begun at least *one* year before it is born. Parents should be enlightened on this subject. They should also be taught to call the dentist and not the physician when the teeth are affected, especially when the teeth are erupting. If the dentist could properly appreciate the importance of his services at this time, he would be able to render invaluable help, and by skill and tact so gain the confidence of the children as to pave the way for early supervision of their teeth. In my opinion it is of more consequence that we give strict attention to the care of children's teeth than adults. It is a matter of great importance that we learn to handle children properly. They can not be treated as adults; their sensitive and childish minds and feelings must be taken into account. It is a fine thing to be able to divert their attention by telling them an interesting story while working for them. A practitioner tells me that he makes it a rule not to work for a child more than fifteen or twenty minutes, and while doing so entertains him with a story such as "how it happened that the hen lost her teeth."—F. M. Parker.

It requires considerable diplomacy to make painful operations for children, and I have resorted to stories, etc. A favorite trick with me is to tell the boy that if he will let me make the operation I will give him a nickle hot from the mint. This I do by polishing a nickle on the laboratory lathe until it is bright and warm and then place it in his hand. It amuses and diverts his mind effectively.—T. N. Inglehart.

